# Flexible Sensor Array Based on Transient Earth Voltage for Online Partial Discharge Monitoring of Cable Termination

**DOI:** 10.3390/s20226646

**Published:** 2020-11-20

**Authors:** Mingshu Zhao, Xiaoyan Cao, Kai Zhou, Yao Fu, Xutao Li, Li Wan

**Affiliations:** 1 Sichuan Energy Internet Research Institute, Tsinghua University, Chengdu 610000, China; zhaomingshu@tsinghua-eiri.org; 2 College of Electrical Engineering, Sichuan University, Chengdu 610065, China; zhoukai@scu.edu.cn (K.Z.); fuyao@stu.scu.edu.cn (Y.F.); wan23li@126.com (L.W.); 3 Electric Power Research Institute, Smart Grid Ningxia Electric Power Company, Yinchuan 750011, China; leeabner@126.com

**Keywords:** partial discharge (PD), online partial discharge (OLPD) monitoring system, cable termination, transient earth voltage (TEV)

## Abstract

Cable termination is a weak point in an underground cable system. The transient earth voltage (TEV) method is an effective and nonintrusive method for estimating the insulation condition of cable termination. However, the practical application of TEV detection is mainly focused on switchgears, generators, and transformers with a flat and conductive shell. A flexible sensor array based on the TEV method is presented for online partial discharge (OLPD) monitoring of the cable termination. Each sensing element is designed with a dual-capacitor structure made of flexible polymer material to obtain better and more stable sensitivity. Based on the electromagnetic (EM) wave propagation theory, the partial discharge (PD) propagation model in the cable termination is built to analyze and verify the rationality and validity of the sensor unit. Some influencing factors are discussed regarding the response characteristics of sensors. Finally, the performance of the sensor array is verified by simulations and experiments. Besides, an OLPD monitoring system is introduced. The monitoring system is composed of the on-site monitoring device and the remote monitoring host. The two parts of the system exchange the data through wireless networks using a wireless communication module. The experiment results show that the monitoring device could supply the PD condition monitoring demand for cable termination.

## 1. Introduction

The cable system is one of the most important and complex components of the infrastructure of power systems, and its safe operation is closely related to the reliability and power supply quality of the power system. In cable systems, cable terminations designed to terminate and connect medium-voltage or high-voltage electrical equipment with cables are more prone to producing insulation defects over time due to long-term environmental, mechanical, and electrical stresses. These insulation defects will excite partial discharge (PD) within strong and inhomogeneous electrical fields, which can eventually lead to high-cost maintenance and long-term outages. Nevertheless, they also can be used as a condition evaluation medium through continuous PD measurement for cable terminations [1,2,3].

PD measurement methods based on electrical, acoustic, chemical, and mechanical phenomena associated with the PD process have been used for many years as nondestructive offline or online techniques for insulation condition evaluation of high-voltage equipment [4,5,6]. These methods include the pulse current method (IEC 60270) [7], HF (3–30 MHz) [8], VHF (30–300 MHz) [9], UHF (300 MHz–3 GHz) method [10,11,12], the transient earth voltage (TEV) method [13,14,15], the acoustic emission method [16,17,18], and the oscillating wave test method [19]. The biggest challenges for continuous PD measurement of cable termination are low-cost and high-sensitivity sensors. Therefore, simple sensors and inexpensive hardware are preferred for implementing an online partial discharge (OLPD) measurement system. The TEV measurement is one of the most promising PD detection methods because of its high sensitivity, wide frequency band, easy installation, high anti-interference performance, small size, and, most importantly, low cost. However, the method is mainly used for PD detection on the metal shell surface of the switchgear or transformers [13,20]. For the cable accessories, HFCTs have been used for PD detection for many years [8,21]. HFCT sensors measure high-frequency pulse currents through magnetic induction methods. PD signals are highly susceptible to interference from the environmental electromagnetic field and current from the grounding wire, and advanced denoising technologies must be considered.

Because the surface of cable termination is semi-conductive, it is possible to use the TEV method for PD detection of cable termination. And because the cable termination is different from switchgear or transformers, the TEV detection principle and influencing factors are also different. For example, the cable termination surface is curved and semi-conductive, while the switchgear surface is flat and conductive. Moreover, given the difference in size, structure, and scale of cable termination and switchgear, the PD propagation path and sensor design are also different. Therefore, a flexible and low-cost TEV sensor is desirable for OLPD monitoring of cable termination.

In this paper, a flexible TEV sensor array is designed, discussed, and verified through an EM wave propagation model. Some PD measurements using the proposed sensor array are performed under laboratory conditions, and the results are analyzed. Additionally, an OLPD monitoring system based on the TEV sensor array is presented for common cable termination.

## 2. Detection Principle and Design of TEV Sensor

### 2.1. Propagation Model of PD in Cable Termination

**Figure 1:**
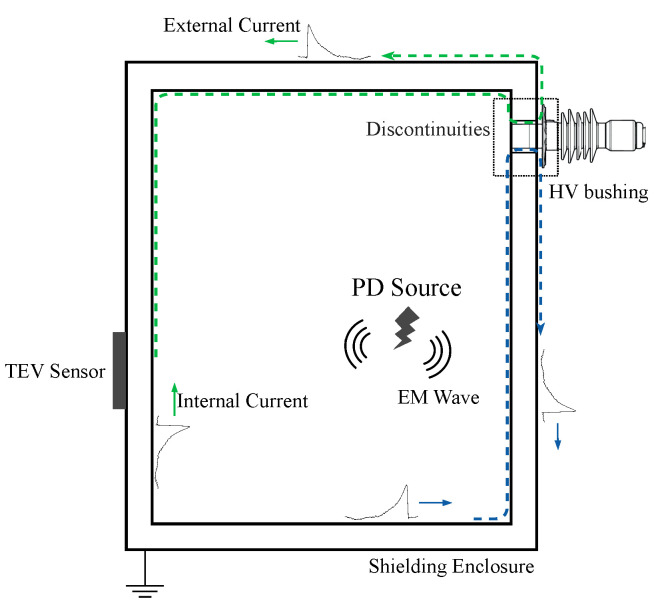
The principle of transient earth voltage (TEV) signal propagation. When partial discharge (PD) occurs inside electrical equipment (simplified to a box), EM waves are emitted. The EM wave leaking from dielectric discontinuities may propagate to the ground through the shield’s outer surface in the form of locally raised voltages (dashed lines). These voltages can be obtained with capacitive sensors and recorded as TEV signals.

When a PD occurs, EM waves propagate away from the discharge site as transient voltage. Due to the skin effect, the transient voltage inside the metalwork cannot be detected directly from the outer surface. However, the EM wave can propagate into free space where there is an electrical discontinuity in the metalwork, which can generate a series of transient voltage spikes on the surface of the surrounding metalwork [20,22], as shown in Figure 1. These spikes are called TEVs and were first proposed by Dr. John Reeves in 1974 [23]. Generally, the PD detection based on the TEV method has mainly been used for switchgear with metal shells.

**Figure 2:**
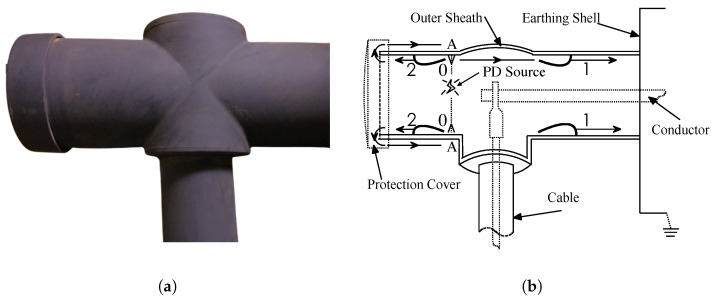
Photograph of a cable termination and corresponding schematic diagram of EM wave propagation path on the cable termination surface. (**a**) Photograph of a 35 kV/600 A elbow cable termination. (**b**) EM wave propagation path on the inner and outer surface of the cable termination.

For the cable termination shown in Figure 2a, EM waves associated with PD propagating along the inner surface of the sheath could transmit to the outer surface (as shown in Figure 2b) from the discontinuous junction between the outer sheath and the cable conductor. These propagated EM waves pass through the cable termination surface to the ground in the form of a locally increased voltage, which can be obtained with a capacitive sensor and recorded as TEV signals [13]. However, due to differences in materials, structures, shapes of terminations, and other factors, the EM propagation process on the cable termination surface is different from that of a switchgear or a transformer [13,20].

Because the propagation characteristics of the EM wave in semiconductors are different from those in conductors, the sensor is a critical part of the TEV measurement system. It is responsible for capturing the electrical signals generated by PD on the cable termination surface. To quantify the sensitivity of the sensor and design an effectively matched filter bank [24], it is necessary to measure the local impedance and establish a PD analysis model based on the traveling wave theory. This paper uses the electromagnetic transients program (EMTP) tool to build a simulation model based on a 35 kV cable termination (Figure 2a).

With an insulation defect inside the cable termination shown in Figure 2b, a PD occurs. Since the semiconductor coating on the cable termination is isotropic, EM waves generated by the PD will propagate through the surface in all directions. To simplify the description, the EM wave propagation process from the discharge point to the inner surface of the sheath is recorded as Wave Process 0. When the EM wave reaches the outer sheath, it will induce a current pulse in the semi-conductive material. There are two paths by which the pulse flows to the ground. One pulse flows directly into the ground along the inner surface of the sheath, denoted as Wave Process 1. Another pulse (Wave Process 2) first reaches the end of the cable termination (the protective back cover) along the inner surface, then escapes from the inner surface of the sheath to the semi-conductive coating, and then continues to propagate to the grounding plate along with the coating and finally passes through the ground plate and ground wire to the earth.

**Figure 3:**
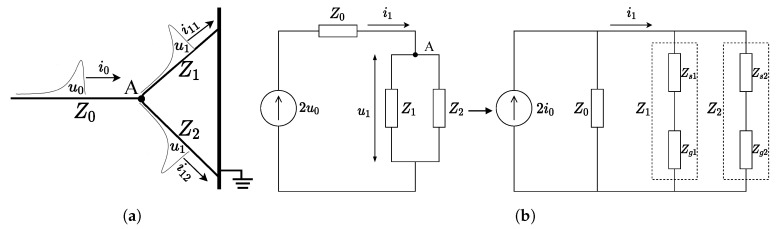
EM wave propagation process and the equivalent circuit for TEV detection. (**a**) EM wave propagation process. (**b**) Equivalent circuit of the TEV detection.

Through the aforementioned analysis, the EM wave propagation of the cable termination can be expressed as Wave Processes 0, 1, and 2, and the transmission line model can be used to address the wave process. The position where the EM wave reaches the inner surface of the outer sheath is marked as Node A, and $Z_{0}$, $Z_{1}$, and $Z_{2}$ represent the equivalent impedances of wave processes 0, 1, and 2, as shown in Figure 3a. According to the lumped-parameter equivalent circuit analysis [25], the EM wave propagation process of the cable termination can be simplified as in Figure 3b. Along the $Z_{1}$ direction, current pulses will pass through two media, the inner surface of the cable termination and the grounded steel plate. The corresponding wave impedances of these two media are recorded as $Z_{s1}$ and $Z_{g1}$, where $Z_{1}=Z_{s1}+Z_{g1}$. Similarly, $Z_{2}=Z_{s2}+Z_{g2}$, which represents the equivalent wave impedance from the inner surface to the protective back cover to the ground electrode.

In the nonintrusive PD test, the outer surface of the cable termination is the ideal monitoring site. In fact, when the PD pulse is detected on the outer surface through this model, the TEV is the voltage on $Z_{g2}$. Moreover, the current flowing through the cable termination surface can be marked as $i_{1}$:

(1) $$i_{1}=\frac{2i_{0}Z_{0}}{\left(Z_{0}+\frac{Z_{1}Z_{2}}{Z_{1}+Z_{2}}\right)}.$$

Thus, the voltage on $Z_{g2}$ (TEV) can be written as

(2) $$U_{TEV}=i_{1}\cdot \frac{Z_{1}Z_{2}}{Z_{1}+Z_{2}}\cdot \frac{Z_{g2}}{Z_{2}}.$$

According to the preceding analysis, a capacitive sensor can measure the TEV produced by the internal PD on the cable termination surface. Because of the small volume of the cable termination, the attenuation of the signal is small, and the amplitude of the TEV is large. Therefore, the capacitive sensor has higher sensitivity with which to capture this TEV signal.

### 2.2. PD Source Model

Due to the characteristics of conventional dielectrics, the current pulse of the PD can be expressed as a Gaussian signal source [26,27], as described in Equation (3):

(3) $$I\left(t\right)=I_{0}\exp\left(-\frac{{\left(t-t_{0}\right)}^{2}}{2\sigma^{2}}\right),$$

where $I_{0}$ is the peak of the current pulse, σ is the standard deviation, and the full width at half maximum (FWHM) of the pulse is $2\sqrt{2\ln2}\sigma \approx 2.355\sigma$. According to Equation (3), the discharge capacity can be expressed by *Q* in Equation (4):

(4) $$Q=\int I\left(t\right)dt=\sqrt{2\pi }I_{0}\sigma ,$$

where *Q* is affected by $I_{0}$ and σ.

As excitation, a Gaussian-shaped current pulse can be used as a discharge source. The current is forced into a non-conductive medium, and the pulse establishes an electrostatic field because space charges are left after the current stops flowing. This is similar to putting the PD in a space-charge-free dielectric material driven by an external electric field [28].

### 2.3. TEV Sensor on a Cable Termination

The pulse duration of the PD could vary from a few nanoseconds to hundreds of nanoseconds. The UHF method has been widely used for PD detection due to its leading advantages, such as low EM interference and high signal-to-noise ratio [29]. However, this method requires a high sampling rate, so that the hardware cost may be huge for processing and storing such a large amount of data for the power distribution network [30,31]. Therefore, considering the characteristics of low cost, high sensitivity, wide frequency band, easy installation, and indirect contact detection, we adopt a capacitive sensor to capture TEV pulses [13].

**Figure 4:**
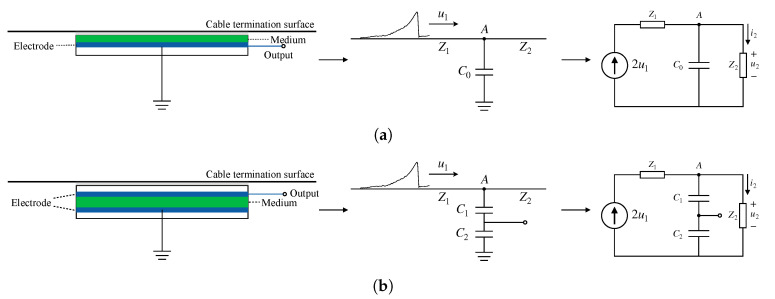
Schematic diagram of traditional and innovative TEV sensors and their simplified traveling wave models. (**a**) Traditional single capacitance sensor. (**b**) Innovative dual capacitance sensor.

In previous reports [13,14,15], the sensing elements used for TEV detection were carefully designed for metal-enclosed switchgear and transformers [13,20], which are only suitable for detecting on a flat surface. To adapt to the curved surface of the cable termination and obtain the largest coupling area to improve detection sensitivity, we designed a fully flexible capacitive TEV sensor made of flexible polymer materials, for example, polyimide film. This design makes the sensing element thickness between 30 μm and 50 μm, which can eliminate the series resistance of the capacitor and greatly reduce the surface resistance of the electrode. For the traditional TEV sensor shown in Figure 4a, assuming that a square wave reaches Node A through Line 1 and bypasses the capacitor $C_{0}$, it continues to propagate on Line 2 after passing through Node A. It can be seen that $u_{c}=u_{2}$. Thus there is a loop equation:

(5) $$2u_{1}=\left(i_{c}+i_{2}\right)Z_{1}+i_{2}Z_{2}=\left(Z_{1}+Z_{2}\right)i_{2}+CZ_{1}Z_{2}\frac{di_{2}}{dt}.$$

Solve the preceding formula to get

(6) $$\left\{\begin{array}{c} i_{2}=\frac{2Z_{1}}{Z_{1}+Z_{2}}i_{1}\left(1-e^{-\frac{t}{\tau_{C}}}\right)\\ u_{2}=\frac{2Z_{2}}{Z_{1}+Z_{2}}u_{1}\left(1-e^{-\frac{t}{\tau_{C}}}\right), \end{array}\right.$$

where $\tau_{C}=CZ_{1}Z_{2}/\left(Z_{1}+Z_{2}\right)$, and

(7) $$\frac{du_{2}}{dt}=\frac{2Z_{2}}{Z_{1}+Z_{2}}u_{1}\frac{1}{\tau_{C}}e^{-\frac{t}{\tau_{C}}}=\frac{2u_{1}}{Z_{1}C}e^{-\frac{t}{\tau_{C}}}.$$

Given that the voltage on the capacitor cannot change suddenly, the square wave becomes an exponential wave after bypassing the capacitor, and the leading edge of the refracted wave passing through Node A can only gradually increase during the process of capacitor charging. Therefore, when the TEV sensor is attached to the surface of the device, the rising edge of the pulse is flattened, and the high-frequency component in the wave is attenuated. In this way, the TEV sensor behaves like an RLC second-order low pass filter. R represents the series and parallel resistance, C represents the coupling capacitance, and L stands for the sensor inductance and the high-frequency signal cable inductance. Normally, the total inductance L can be ignored, and the filter can be further simplified as an RC low pass filter. When the capacitance C is too small, the influence of inductance needs to be considered. According to the aforementioned analysis, the most important part of the TEV sensor is the coupling capacitance. Therefore the coupling area and pole distance are major factors affecting the sensor’s performance. Hence, the small coupling area due to the curved surface and the unstable sensitivity due to the installation process are critical problems when applying traditional TEV sensors to cable terminations.

According to the traditional TEV detection principle, the TEV sensor and the cable termination surface forms a flat capacitance $C_{0}$ (called mutual capacitance), as shown in Figure 4a. When the sensor is manufactured, the material permittivity (ϵ) and surface area (*S*) parameters are fixed, and the only variable factor is the distance ($d_{0}$) between the electrodes (the sensor and the surface of cable termination). The capacitance can be represented as $C_{0}=\epsilon S/d_{0}$. When the distance between the TEV sensor and the cable termination surface changes, in other words, $d_{0}$ will change with Δd:

(8) $$C=C_{0}\pm \Delta C=\frac{\epsilon S}{d_{0}\pm \Delta d}=\frac{C_{0}}{1\pm \frac{\Delta d}{d_{0}}},$$

where *C* can be further represented by Taylor series as:

(9) $$\begin{array}{cc} C & =\left\{\begin{array}{c} C_{0}\sum_{n=0}^{\infty }{\left(-1\right)}^{n}{\left(\frac{\Delta d}{d_{0}}\right)}^{n}\\ C_{0}\sum_{n=0}^{\infty }{\left(\frac{\Delta d}{d_{0}}\right)}^{n} \end{array}\right. \end{array}$$

(10) $$\begin{array}{cc} & =C_{0}\left[1\pm \frac{\Delta d}{d_{0}}+{\left(\frac{\Delta d}{d_{0}}\right)}^{2}\pm {\left(\frac{\Delta d}{d_{0}}\right)}^{3}+\cdots \right]. \end{array}$$

**Figure 5:**
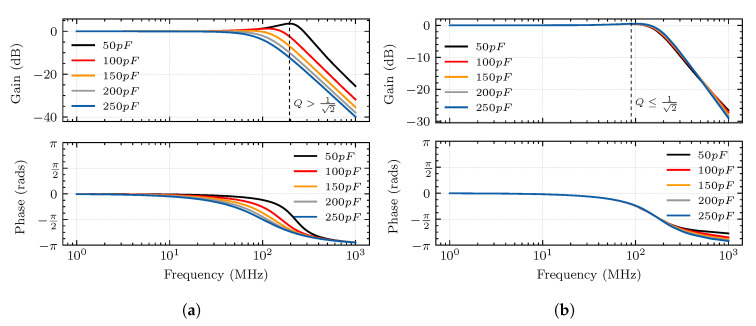
Simulated frequency responses of traditional and innovative TEV sensors at different mutual capacitances. (**a**) The sensitivity of the traditional TEV sensor greatly depends on mutual capacitance. (**b**) The sensitivity of the innovative TEV sensor is less dependent on mutual capacitance.

The capacitance can be determined as $C_{0}\left(1\pm \Delta d/d_{0}\right)$ when $\Delta d\ll d_{0}$, which means the capacitance is approximately linearly related to the distance between the TEV sensor and the cable termination surface. However, Δd is determined by the sensor installation process, as with the pressure on the surface of termination, so it is easy to destroy the linear relationship and lead to decreased sensitivity, as shown in Figure 5a.

To reduce the influence during the installation process and improve the TEV sensor’s sensitivity, we designed the sensor as a dual capacitance series inspired by the differential structure, as shown in Figure 4b. The sensor itself is designed as a complete capacitor with two electrodes (called self-capacitance $C_{2}$). During the detection process, the electrode at one end of the medium is attached to the cable termination surface. To prevent the sensor electrode from making direct contact with the cable termination surface and causing a discharge to the ground, we sealed the entire sensor with a plastic film and only led out the signal line from the electrodes. The electrode near the cable termination was used as a signal output terminal, and the other electrode was grounded. When the sensor was attached to the cable termination surface, the air gap between the output electrode and the cable termination surface constituted another capacitance (called mutual capacitance $C_{1}$).

Same as the previous analysis method, when the distance between the sensor and the cable termination surface changes Δd:

(11) $$C=\frac{\left(C_{1}\pm \Delta C\right)C_{2}}{\left(C_{1}\pm \Delta C\right)+C_{2}}=\frac{C_{1}C_{2}}{C_{1}+\left(1\pm \frac{\Delta d}{d_{0}}\right)C_{2}}=\frac{\frac{C_{1}C_{2}}{C_{1}+C_{2}}}{1\pm \frac{C_{2}}{C_{1}+C_{2}}\cdot \frac{\Delta d}{d_{0}}},$$

where *C* can be further represented by Taylor series as:

(12) $$\begin{array}{cc} C & =\left\{\begin{array}{c} \frac{C_{1}C_{2}}{C_{1}+C_{2}}\sum_{n=0}^{\infty }{\left(-1\right)}^{n}{\left(\frac{C_{2}}{C_{1}+C_{2}}\cdot \frac{\Delta d}{d_{0}}\right)}^{n}\\ \frac{C_{1}C_{2}}{C_{1}+C_{2}}\sum_{n=0}^{\infty }{\left(\frac{C_{2}}{C_{1}+C_{2}}\cdot \frac{\Delta d}{d_{0}}\right)}^{n} \end{array}\right. \end{array}$$

(13) $$\begin{array}{cc} \; & =\frac{C_{1}C_{2}}{C_{1}+C_{2}}\left[1\pm \left(\frac{C_{2}}{C_{1}+C_{2}}\cdot \frac{\Delta d}{d_{0}}\right)+{\left(\frac{C_{2}}{C_{1}+C_{2}}\cdot \frac{\Delta d}{d_{0}}\right)}^{2}\pm {\left(\frac{C_{2}}{C_{1}+C_{2}}\cdot \frac{\Delta d}{d_{0}}\right)}^{3}+\cdots \right]. \end{array}$$

The capacitance can be determined as $C_{1}C_{2}/\left(C_{1}+C_{2}\right)\cdot \left(1\pm C_{2}\Delta d/\left[\left(C_{1}+C_{2}\right)d_{0}\right]\right)$ when $\Delta d\ll d_{0}$, which means the capacitance not only determined by the distance of sensor and cable termination surface, but also correlated with the self-capacitance $C_{2}$.

In analyzing the frequency responses of the traditional and innovative TEV sensors shown in Figure 5, it becomes clear that the sensor circuit behaves like an RLC low pass filter, where the quality factor can be regarded as $Q=1/\left(\omega_{0}RC\right)$. The quality factor determines the amount of peaking for the filter’s frequency response, and there’s no peaking for $Q\leq 1/\sqrt{2}=0.707$. According to Figure 4, the innovative sensor’s quality factor is generally not too large because its overall capacitance is composed of self-capacitance and mutual capacitance. In contrast, the quality factor of traditional sensors is only determined by mutual capacitance, so the range of variation is wider, and hence the amount of peaking is larger when the capacitance value is smaller. Besides, if we ignore the total inductance, the sensor circuit can be further simplified into an RC low-pass filter. The cut-off frequency $f_{c}=1/\left(2\pi RC\right)$ of the traditional TEV sensor is greatly dependent on the mutual capacitance, whereas the cut-off frequency of the innovative sensor is determined by the mutual capacitance and self-capacitance. If the self-capacitance is small enough, such as less than the mutual capacitance, the changes of mutual capacitance will decrease. Therefore, using an innovative TEV sensor design can effectively reduce the frequency band degradation problem caused by the sensor installation process and achieve higher and more stable detection sensitivity.

## 3. Response Characteristics of the TEV Sensor

### 3.1. Effect of Capacitance on Response Characteristics

According to the previous analysis, after the sensor is installed on the cable termination surface, a dual-capacitance-series detection model is formed, and the TEV signal on the surface is obtained from the output terminal by dividing the voltage of $C_{1}$ and $C_{2}$. However, the resistivity of semiconductors is much higher than that of conductors. From the perspective of circuit analysis, the detection point’s output impedance is much greater than 0, so when the sensor capacitance is attached to the cable termination surface, it will affect the ground impedance of the detection point, thereby changing the distribution of the TEV. Therefore, it is important to investigate the effect of different capacitances on the output response.

Like the traditional TEV sensor, the innovative sensor equivalent circuit (shown in Figure 4b) is similar to a low-pass filter. When the pulse signal passes through this circuit, the high-frequency components are filtered out, so the amplitude of the signal and the steepness of the pulse edge decrease. Therefore, one way to increase the sensor’s detection frequency band is to reduce the series capacitance of $C_{1}$ and $C_{2}$.

**Figure 6:**
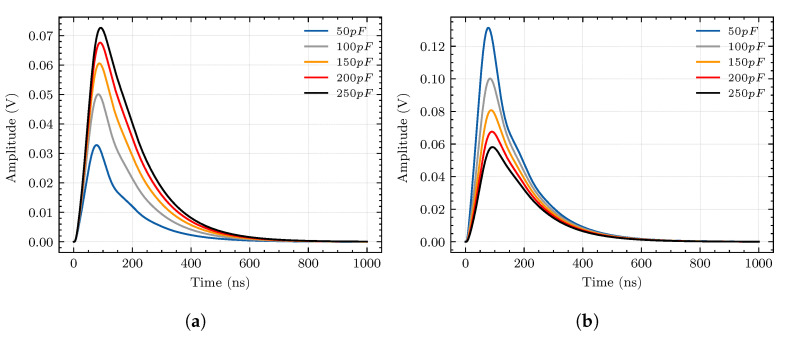
Sensor responses of different capacitances. (**a**) Mutual capacitance *C*_1_ is positively correlated with sensor response. (**b**) Self-capacitance *C*_2_ is negatively correlated with sensor response.

We know that $C_{1}$ is related to the fit degree between the sensor and the surface of the cable termination through the previous analysis. The main factor affecting its value is the pole distance *d*, where $C_{1}$ increases with the decrease in *d*. To investigate the effect of the mutual capacitance $C_{1}$ on the performances of the sensor, we keep $C_{2}$ unchanged and set the value of $C_{1}$ to 50 pF, 100 pF, 150 pF, 200 pF, and 250 pF for simulation; the response appears in Figure 6a.

The self-capacitance $C_{2}$ of the sensor is different from the mutual capacitance $C_{1}$. It is fixed after the sensor is manufactured. Therefore, the self-capacitance $C_{2}$ must be adjusted before manufacturing the sensor. Similar to the previous analysis method, we keep $C_{1}$ unchanged and set the value of $C_{2}$ to 50 pF, 100 pF, 150 pF, 200 pF, and 250 pF, respectively. The simulation result of the sensor output pulse is shown in Figure 6b.

According to the simulation results, the sensor’s output amplitude is positively correlated with the capacitance of $C_{1}$ while being negatively correlated with the capacitance of $C_{2}$. This indicates that the smaller the gap between the sensor and the cable termination surface (the larger the mutual capacitance $C_{1}$), and the smaller the self-capacitance of the sensor ($C_{2}$), the higher the sensitivity of the sensor. However, there exists a trade-off between sensor sensitivity and stability. The small self-capacitance will lead to unstable performance according to Equation (12) and Figure 5.

The changes in response amplitude caused by changes in $C_{1}$ and $C_{2}$ are different. From 50 pF to 250 pF, $C_{1}$ causes a change of 40 mV, while $C_{2}$ causes a change of nearly 80 mV. In summary, the value of self-capacitance $C_{2}$ needs to be reduced when manufacturing the sensor, the sensor should be as close as possible to the measured surface (increase $C_{1}$) during the measurement process, and $C_{2}$ is the dominant one to improve the sensitivity of the sensor.

### 3.2. Influence of Installation Position of the Sensor

**Figure 7:**
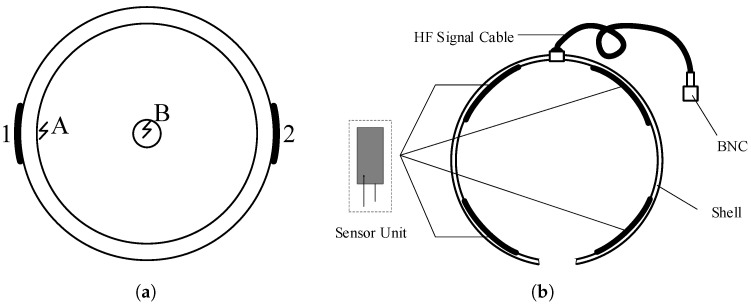
Schematic diagram of the PD source location and TEV sensor array structure. (**a**) Schematic diagram of PD sources at different locations. (**b**) Structure of the sensor array with four sensor elements.

**Figure 8:**
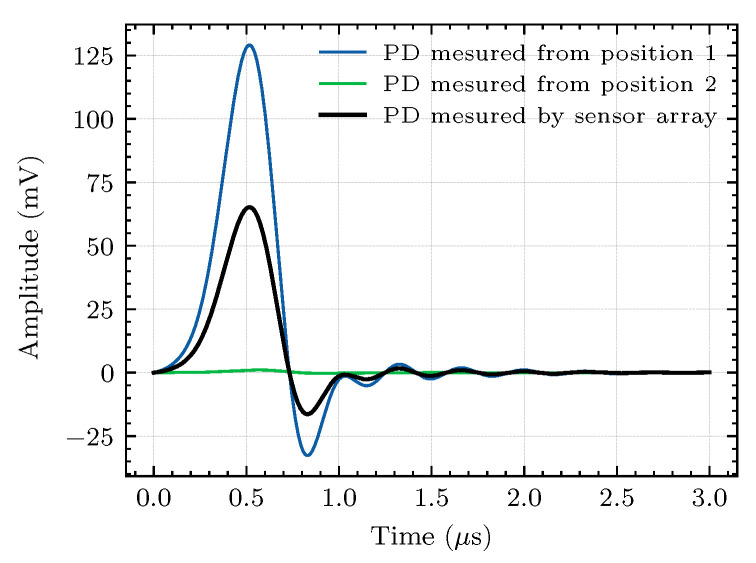
Simulated PD responses of the sensor array and the single sensor at different detection positions.

Different PD pulse propagation distances have different impedances because of different propagation paths. If the discharge occurs near the internal conductor (Point B as shown in Figure 7a), the output response should be similar, regardless of whether the sensor is in Position 1 or Position 2 because the distance from the center (Point B) to the outer surface is equal. However, except for this ideal internal discharge location, most of the discharges occur at random locations. A more common simulation situation is discussed as follows. In this case, the discharge caused by the insulation defect occurs between the main insulator surface and the outer sheath, which means that the discharge source is located at Point A in Figure 7a. Under this situation, the pulse captured in Position 1 is much stronger than that in Position 2, as shown in Figure 8. To improve the generalization performance, a sensor array containing four sensor elements is employed to capture TEV signals. The structure of the sensor array is presented in Figure 7b. The output waveform of the sensor array is the ensemble average value of each sensing element, as shown in Figure 8. Generally speaking, the sensor array is more robust than a single sensor for capturing the PD signal at any position. Besides, the different waveform propagation paths between the PD source and the sensor will cause different signal attenuation, resulting in different signal amplitudes. Therefore, the sensor should be placed as close to the protection cover as possible to reduce the propagation distance.

### 3.3. Experimental Verification for the Sensor

**Figure 9:**
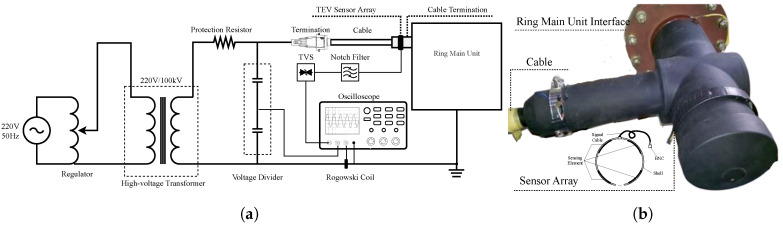
Schematic diagrams of experiment arrangement. (**a**) Schematic representation of the test setup showing the physical arrangement. (**b**) The cable termination and flexible TEV sensor array in a laboratory environment. A 1-centimeter-long metal particle is installed inside the cable termination and about 2 centimeters from the protective cover as an artificial defect.

To verify the performance of the developed TEV sensor array, an EMTP simulation model based on the 35 kV elbow cable termination was established, as shown in Figure 2, and PD waveforms were generated based on Gaussian current pulses (as Equation (3)). Simultaneously, an actual experiment platform consisting of a 3-meter-long 35 kV underground power cable and 2 terminations with artificial defects was arranged in the high-voltage laboratory for real-time analysis, as described in Figure 9. The TEV sensor collects PD waveforms under test conditions and compares them with simulation data to verify the rationality and effectiveness of the model and sensor. Because a simulation model is established based on the propagation path of EM waves, the EM propagation characteristics of different materials are the main factors in the reproducibility of the simulation and experiment. With this in mind, model parameters such as wave impedance and wave speed were first gathered based on the cable termination material being tested.

**Figure 10:**
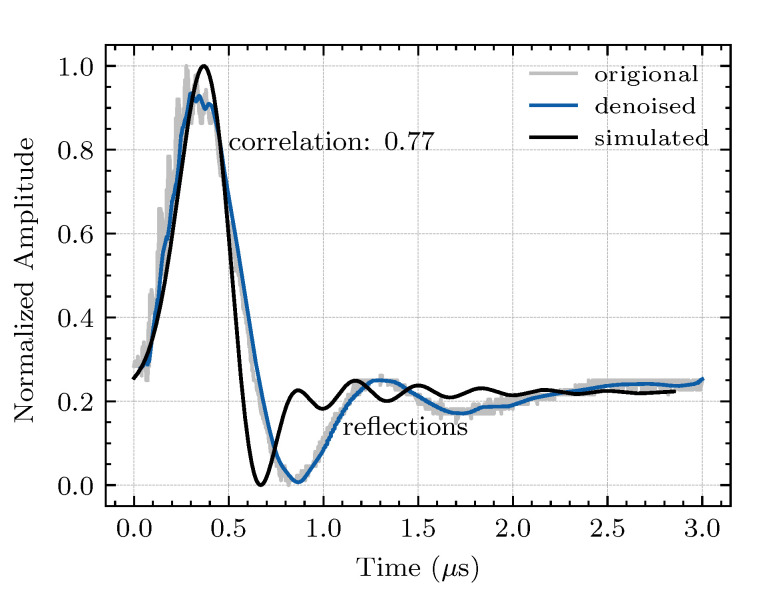
Experimental and simulated PD waveforms. The wavelet-denoised experimental waveform has a strong correlation with the simulated waveform.

The propagation of the simulated EM wave through the cable termination surface can be detected through the voltmeter-to-ground in the model (as shown in Figure 2), and a data acquisition unit with a maximum sampling frequency of 40 MHz can collect the experimental PD waveform captured by the TEV sensor. The simulated response and measured signal are shown in Figure 10. The measured waveform (incorporating the propagation velocities and the amplitudes depending on the impedances) is highly correlated with the simulation waveform. Therefore, simulation models are implemented to automate selection of the optimal sensor installation location and the best sensor parameters.

**Figure 11:**
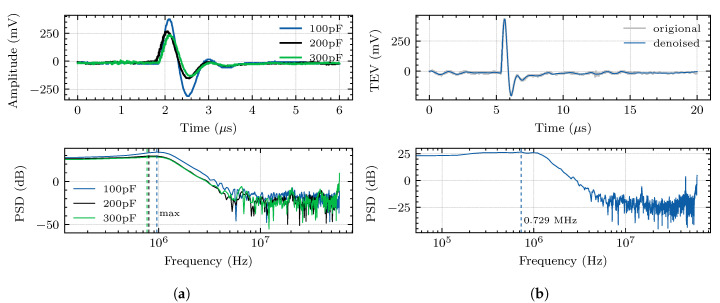
Time and frequency waveforms of PD within the test environment. (**a**) The measured PD waveforms and corresponding power spectral density (PSD) of different self-capacitance *C*_2_ under laboratory conditions. (**b**) The time-resolved PD pulse waveform and its corresponding PSD measured by the designed TEV sensor array.

The self-capacitance of the sensor can be changed by the thickness of the dielectric material. To investigate the influence of self-capacitance on pulse rise and attenuation, we designed three sensors with different self-capacitance: 100 pF, 200 pF, and 300 pF. Figure 11a shows the waveforms captured by the sensors with different self-capacitance based on the experiment in Figure 9. It can be seen from the figure that the main frequency of the PD waveform collected by the TEV sensor decreases as the self-capacitance increases. Therefore, if the self-capacitance is too small, the hardware’s cost will increase due to the increased sampling rate. On the contrary, if the self-capacitance is too large, the detection sensitivity will reduce due to the decrease in the signal amplitude. Figure 11b shows the TEV signal measured by the TEV sensor array in a laboratory environment, as shown in Figure 9. The main pulse of the TEV signal is about 0.9 μs, and its main frequency spectrum is about 0.73 MHz. According to model analysis and experimental verification, the flexible sensor array based on the TEV measurement can effectively obtain PD information with low cost and high sensitivity.

## 4. OLPD Monitoring System Based on Flexible TEV Sensor Array

The OLPD monitoring system is a sophisticated system that can automatically detect, diagnose, locate, and evaluate the aging severity and failure risk of all types of in-service HV assets. The two main objectives of implementing an OLPD monitoring system are to improve the facility’s safety and minimize unplanned outages caused by HV insulation failure [32]. The significant reduction in the cost of integrated circuits and memory and the tremendous progress in microprocessors, hardware, and software used to process PD data, make OLPD more cost effective [33]. This benefit drives OLPD monitoring technology to be more widely used in power equipment fault diagnosis, as with transformers, switchgear, cable joints, and generator stators windings [11,21,34].

**Figure 12:**
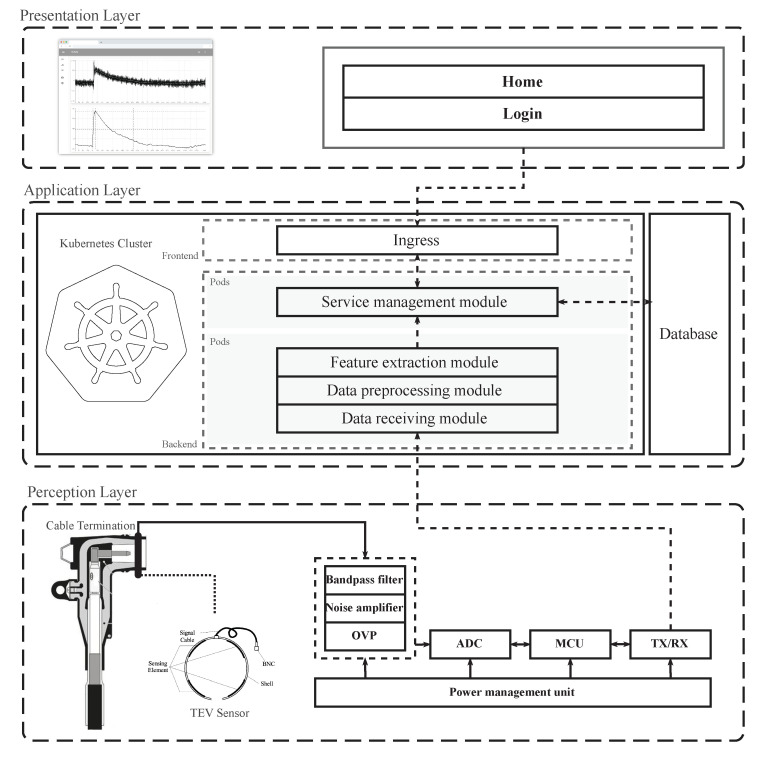
Overall design architecture of the OLPD monitoring system based on the TEV sensor.

The workflow of the cable termination PD monitoring system is shown in Figure 12. First, the signals from one or more sensors are filtered and amplified before being detected and digitized. The digitized signal from the monitoring equipment after pre-processing is then concentrated to the central server through a local area network. Many microservices are running in the server for storing, analyzing, accessing data, and setting alarm levels. Finally, a neuron-based or tree-based intelligent software agent is used to extract patterns from the data and provide meaningful information about the characteristics of the PD source [35,36,37,38]. Furthermore, times of arrival (TOA) or cross-correlation algorithms can be implemented to locate the PD source [39].

The monitoring system contains three parts: the perception layer, for collecting and transmitting the PD waveform; the application layer, for analyzing and storing the data; and the presentation layer, for displaying the analysis results.

The perception layer is composed of the flexible TEV sensor array, the high-frequency signal line, the microprocessor, the power supply module, the analog signal processing module, the AD conversion module, and the wireless signal transmission module. Given that the phase of the inner PD usually appears in the first and third quadrants of power frequency voltage [40], the phase-resolved partial discharge (PRPD), which visualizes occurrence of PD activities in reference to the phase of applied AC voltage [8,12,41], is used for the PD level assessment of the cable termination. A power frequency cycle is used as the duration of every sampling, and the amplitude, phase, and times of discharges that happen during this time will be wholly recorded. After every sampling, the data in the cache of the data acquisition module are immediately taken away by the microprocessor’s storage, and the cache is freed up. In this way, the cache will be free before every sampling, which could greatly reduce the cost of the data acquisition cache and CPU utilization. Moreover, data are processed before being transmitted to the central server to avoid consuming too much transmission bandwidth.

The application layer is a server cluster managed by Kubernetes. Many microservices are running inside the cluster to receive, store, analyze, and display the PD waveform. The presentation layer is the web interface for the final users. Through the website, users can get real-time, historical, or statistical PD information.

## 5. Conclusions

A new type of flexible TEV sensor array for PD detection of cable termination is introduced, and an OLPD monitoring system based on this sensor array is proposed. Experiments and simulations methods are established to verify the rationality and effectiveness of the sensor and system. The summary and main conclusions are as follows.

- The TEV method is proposed to detect the PD of the cable termination, and the EM wave propagation model of the cable termination was established for the TEV sensor design.
- A flexible TEV sensor array is presented to adapt to the curved surface of cable termination and obtain the largest coupling area. Each sensing element is designed as a dual-capacitance structure to reduce the sensitivity degradation problem caused by the sensor installation process and achieve higher and more stable detection sensitivity.
- An OLPD monitoring system based on the flexible TEV sensor array is established. It is composed of the perception layer, the application layer, and the presentation layer.

## References

[1] Eigner A., Rethmeier K. (2016). An overview on the current status of partial discharge measurements on AC high voltage cable accessories. IEEE Electr. Insul. Mag..

[2] Gulski E., Smit J.J., Wester F.J. (2005). PD knowledge rules for insulation condition assessment of distribution power cables. IEEE Trans. Dielectr. Electr. Insul..

[3] Mousavi Gargari S., Wouters P.A.A.F., van der Wielen P.C.J.M., Steennis E.F. (2011). Partial discharge parameters to evaluate the insulation condition of on-line located defects in medium voltage cable networks. IEEE Trans. Dielectr. Electr. Insul..

[4] Sikorski W., Walczak K., Gil W., Szymczak C. (2020). On-Line Partial Discharge Monitoring System for Power Transformers Based on the Simultaneous Detection of High Frequency, Ultra-High Frequency, and Acoustic Emission Signals. Energies.

[5] Wagenaars P. (2010). Integration of Online Partial Discharge Monitoring and Defect Location in Medium-Voltage Cable Networks. Ph.D. Thesis.

[6] Wu M., Cao H., Cao J., Nguyen H., Gomes J.B., Krishnaswamy S.P. (2015). An overview of state-of-the-art partial discharge analysis techniques for condition monitoring. IEEE Electr. Insul. Mag..

[7] IEC (2015). IEC 60270:2000/AMD1:2015, High-Voltage Test Techniques—Partial Discharge Measurements.

[8] Wu J., Rodrigo Mor A., van Nes P.V.M., Smit J.J. (2020). Measuring method for partial discharges in a high voltage cable system subjected to impulse and superimposed voltage under laboratory conditions. Int. J. Electr. Power Energy Syst..

[9] Tian Y., Lewin P.L., Davies A.E., Sutton S.J., Swingler S.G. (2003). Partial discharge detection in cables using VHF capacitive couplers. IEEE Trans. Dielectr. Electr. Insul..

[10] Beura C.P., Beltle M., Tenbohlen S. (2020). Study of the Influence of Winding and Sensor Design on Ultra-High Frequency Partial Discharge Signals in Power Transformers. Sensors.

[11] Judd M.D., Yang L., Hunter I.B.B. (2005). Partial discharge monitoring of power transformers using UHF sensors. Part I: Sensors and signal interpretation. IEEE Electr. Insul. Mag..

[12] Dukanac D. (2018). Application of UHF method for partial discharge source location in power transformers. IEEE Trans. Dielectr. Electr. Insul..

[13] Zhang C., Dong M., Ren M., Huang W., Zhou J., Gao X., Albarracín R. (2018). Partial Discharge Monitoring on Metal-Enclosed Switchgear with Distributed Non-Contact Sensors. Sensors.

[14] Ren M., Dong M., Ren Z., Peng H., Qiu A. (2012). Transient Earth Voltage Measurement in PD Detection of Artificial Defect Models in SF_6_. IEEE Trans. Plasma Sci..

[15] Luo G., Zhang D., Tseng K.J., He J. (2016). Impulsive noise reduction for transient Earth voltage-based partial discharge using Wavelet-entropy. IET Sci. Meas. Technol..

[16] Lundgaard L.E. (1992). Partial discharge. XIII. Acoustic partial discharge detection-fundamental considerations. IEEE Electr. Insul. Mag..

[17] Lundgaard L.E., Runde M., Skyberg B. (1990). Acoustic diagnosis of gas insulated substations: A theoretical and experimental basis. IEEE Trans. Power Deliv..

[18] Sikorski W. (2019). Development of Acoustic Emission Sensor Optimized for Partial Discharge Monitoring in Power Transformers. Sensors.

[19] Zhu G., Zhou K., Zhao S., Li Y., Lu L. (2020). A Novel Oscillation Wave Test System for Partial Discharge Detection in XLPE Cable Lines. IEEE Trans. Power Deliv..

[20] Itose A., Kozako M., Hikita M. Partial discharge detection and induced surface current analysis using transient earth voltage method for high voltage equipment. Proceedings of the 2016 International Conference on Condition Monitoring and Diagnosis (CMD).

[21] Wu R., Chang C. (2011). The Use of Partial Discharges as an Online Monitoring System for Underground Cable Joints. IEEE Trans. Power Deliv..

[22] Yoshizumi H., Koga T., Kozako M., Hikita M., Fujii Y., Nakamura Y., Cho H. Grounding effect on transient earth voltage signal induced by partial discharge in metal box model. Proceedings of the 2017 International Symposium on Electrical Insulating Materials (ISEIM).

[23] Davies N., Tian Y., Cheung J., Tang Y., Shiel P. Non-intrusive partial discharge measurements of MV switchgears. Proceedings of the 2008 International Conference on Condition Monitoring and Diagnosis.

[24] Veen J., van der Wiellen P.C.J.M. (2003). The application of matched filters to PD detection and localization. IEEE Electr. Insul. Mag..

[25] Rizk F., Trinh G. (2018). High Voltage Engineering.

[26] Boggs S.A., Stone G.C. (1982). Fundamental Limitations in the Measurement of Corona and Partial Discharge. IEEE Trans. Electr. Insul..

[27] Tang J., Zhou Q., Tang M., Xie Y. (2007). Study on mathematical model for VHF partial discharge of typical insulated defects in GIS. IEEE Trans. Dielectr. Electr. Insul..

[28] Pommerenke D., Jobava R., Heinrich R. (2002). Numerical simulation of partial discharge propagation in cable joints using the finite difference time domain method. IEEE Electr. Insul. Mag..

[29] Jahangir H., Akbari A., Werle P., Szczechowski J. (2017). UHF PD measurements on power transformers-advantages and limitations. IEEE Trans. Dielectr. Electr. Insul..

[30] Álvarez F., Garnacho F., Ortego J., Sánchez-Urán M. (2015). Application of HFCT and UHF Sensors in On-Line Partial Discharge Measurements for Insulation Diagnosis of High Voltage Equipment. Sensors.

[31] Chai H., Phung B., Mitchell S. (2019). Application of UHF Sensors in Power System Equipment for Partial Discharge Detection: A Review. Sensors.

[32] Renforth L.A., Giussani R., Mendiola M.T., Dodd L. (2019). Online Partial Discharge Insulation Condition Monitoring of Complete High-Voltage Networks. IEEE Trans. Ind. Appl..

[33] Stone G.C. (2005). Partial discharge diagnostics and electrical equipment insulation condition assessment. IEEE Trans. Dielectr. Electr. Insul..

[34] Lloyd B.A., Campbell S.R., Stone G.C. (1999). Continuous on-line partial discharge monitoring of generator stator windings. IEEE Trans. Energy Convers..

[35] Su M.S., Chia C.C., Chen C.Y., Chen J.F. (2014). Classification of partial discharge events in GILBS using probabilistic neural networks and the fuzzy c-means clustering approach. Int. J. Electr. Power Energy Syst..

[36] Krivda A. (1995). Automated recognition of partial discharges. IEEE Trans. Dielectr. Electr. Insul..

[37] Peng X., Li J., Wang G., Wu Y., Li L., Li Z., Ahmed Bhatti A., Zhou C., Hepburn D.M., Reid A.J. (2019). Random Forest Based Optimal Feature Selection for Partial Discharge Pattern Recognition in HV Cables. IEEE Trans. Power Deliv..

[38] Peng X., Yang F., Wang G., Wu Y., Li L., Li Z., Bhatti A.A., Zhou C., Hepburn D.M., Reid A.J. (2019). A Convolutional Neural Network-Based Deep Learning Methodology for Recognition of Partial Discharge Patterns from High-Voltage Cables. IEEE Trans. Power Deliv..

[39] Rao X., Zhou K., Li Y., Zhu G., Meng P. (2020). A New Cross-Correlation Algorithm Based on Distance for Improving Localization Accuracy of Partial Discharge in Cables Lines. Energies.

[40] Sakoda T., Arita T., Nieda H., Ando K., Otsub M., Honda C. (1999). Studies of elastic waves caused by corona discharges in oil. IEEE Trans. Dielectr. Electr. Insul..

[41] Firuzi K., Vakilian M., Phung B.T., Blackburn T.R. (2019). Partial Discharges Pattern Recognition of Transformer Defect Model by LBP HOG Features. IEEE Trans. Power Deliv..

